# Intelligent Optimization of Hard-Turning Parameters Using Evolutionary Algorithms for Smart Manufacturing

**DOI:** 10.3390/ma12060879

**Published:** 2019-03-15

**Authors:** Mozammel Mia, Grzegorz Królczyk, Radosław Maruda, Szymon Wojciechowski

**Affiliations:** 1 Department of Mechanical and Production Engineering, Ahsanullah University of Science and Technology, Dhaka 1208, Bangladesh; mozammelmiaipe@gmail.com; 2 Faculty of Mechanical Engineering, Opole University of Technology, St. Mikołajczyka 5, 45-001 Opole, Poland; 3 Faculty of Mechanical Engineering, University of Zielona Gora, 4 Prof. Z. Szafrana Street, 65-516 Zielona Gora, Poland; r.maruda@ibem.uz.zgora.pl; 4 Faculty of Mechanical Engineering and Management, Poznan University of Technology, 3 Piotrowo St., 60-965 Poznan, Poland; sjwojciechowski@o2.pl

**Keywords:** intelligent optimization, hard turning, surface roughness, cutting temperature, evolutionary algorithm

## Abstract

Recently, the concept of smart manufacturing systems urges for intelligent optimization of process parameters to eliminate wastage of resources, especially materials and energy. In this context, the current study deals with optimization of hard-turning parameters using evolutionary algorithms. Though the complex programming, parameters selection, and ability to obtain the global optimal solution are major concerns of evolutionary based algorithms, in the present paper, the optimization was performed by using efficient algorithms i.e., teaching–learning-based optimization and bacterial foraging optimization. Furthermore, the weighted sum method was used to transform the diverse responses into a single response, and then multi-objective optimization was performed using the teaching–learning-based optimization method and the standard bacterial foraging optimization method. Finally, the optimum results reported by these methods are compared to choose the best method. In fact, owing to better convergence within shortest time, the teaching–learning-based optimization approach is recommended. It is expected that the outcome of this research would help to efficiently and intelligently perform the hard-turning process under automatic and optimized environment.

## 1. Introduction

Smart manufacturing (SM) is regarded as the next generation manufacturing revolution—Industry 4.0 [1,2]. In this technology, the manufacturing system is optimized to the highest level to extract the highest benefits in terms of production economics, quality, and time. Use of advanced technologies such as sensors, smart materials, production and process planning, cloud systems etc., and their interaction with humans determines the success of a smart manufacturing unit [3]. In a nutshell, SM is a technology that allows the process improvement via optimization and exploitation of advanced technologies that establish it as a next generation manufacturing model.

Intelligent optimization of real-life manufacturing systems is key factor to smart manufacturing. The implementation of an intelligent optimization technique in the soft part of manufacturing units facilitates an effective production control. Among prevailing optimization techniques (statistical, neural, evolutionary, machine learning, etc.) the evolutionary methods have recently been employed successfully in various engineering sectors. Traditionally, the effectiveness and efficiency of selecting parameters of machining processes are determined by the trial-and-run method or by the experience of the machine operators [4,5]. As per the requirements of smart manufacturing as in ‘turning’ systems, the cutting parameters i.e., cutting speed, feed rate, and depth-of-cut need to be optimized. This information can be synchronized with the database of the manufacturing unit. In fact, as an upgrade based on instantaneous requirements, the control factors can be optimized in real-time. In this particular segment, the learning capability of modeling methods (i.e., artificial intelligence, machine learning) possesses the potential to enhance requirement-based manufacturing to reduce human intervention [6].

Successful implementation of intelligent/evolutionary methods in the manufacturing realm can be found in literature. For instance, Rao et al. [7] applied the novel teaching–learning-based optimization (TLBO) method to optimize multiple mechanical design problems. In studying the welding of Cr–Mo–V steel, Rao and Kalyankar [8] applied TLBO alongside the Taguchi-based optimization. In another study, Pawar and Rao [9] applied TLBO in abrasive water jet machining, milling, and grinding to optimize the machining parameters. They compared the results of TLBO with other methods of optimization and found better results of TLBO. In another paper, Rao and Kalyankar [10] optimized the parameters of modern machining processes namely the Ultra Sound Machining (USM), Abrasive Jet Machining (AJM), and Wire Electrical Discharge Machining (WEDM) using TLBO. Gupta et al. [11] employed one statistical method (i.e., Response Surface Methodology (RSM)) and one evolutionary method (i.e., Particle Swarm Optimization (PSO)) to optimize the machining parameters. Mukhopadhyay et al. [12] employed an artificial neural network and genetic algorithm for the modeling and optimization of the wire electrical discharge machining process. In addition, they have implemented the hybrid modeling to extract better machining optimization results. Kim and Lee [13] optimized the induction-assisted milling process using finite element analysis, signal-to-noise ratio, and analysis of variance. The optimized responses were surface roughness, tool wear, and surface roughness in selected machining environment.

In high-performance precision engineering application, the quality of products produced by turning process is evaluated by the roughness parameters of machined surface [14]. The increased pressure from the industries to produce parts with very low surface-roughness values forces researchers to find ways of reducing surface roughness. Among many alternatives, optimization of process parameters can refine the surface roughness value. This means the appropriate parameter settings of control factors can generate surfaces with a surface roughness value that is lower than the roughness found in conventional processes and/or hard machining. Hybridization of PSO–bacteria foraging optimization (BFO) was reported in the optimization of additive manufacturing parameters for the fused deposition modeling Raju et al. [15]. As can be seen, the advanced computational methods for optimization have been reported in multifarious sectors such as welding, additive manufacturing, machining, modern machining processes, etc. However, very few articles reported the adoption of evolutionary algorithms for hard turning.

Besides the surface roughness parameter, the overall machining outcomes are largely influenced by the cutting temperature. The intensive friction induced by the plastic deformation during the chip generation cause the mechanical energy to be transformed into heat energy. As such, the chip–tool interface and work–tool interface temperature rises. An increase in temperature results in the expedited wear rate of tool; hence, the machining economy is compromised. Also, the premature failure or sudden breakage of the tool can result in the adherence of tool broken debris onside the machine surface—rejection of the product. In that perspective, controlling of cutting zone temperature is inevitable.

Use of cooling/lubricating agents during cutting is widely accepted as a temperature-controlling system and surface-quality improver. However, the practice of coolant/lubricant is costly, and it causes serious harm to the environment and to human health. In that respect, the clean and sustainable manufacturing system that must be a smart manufacturing system too can be attained by the intelligent optimization of process parameters so that the benefits of using coolant/lubricant are alternatively achieved by the adoption of optimum control factor settings.

In the current paper, the control factors of hard-turning operation are optimized using intelligent evolutionary algorithms. The optimization is performed using two methods: (i) teaching–learning-based optimization and (ii) bacterial foraging optimization. Finally, these two methods are compared between themselves to select the best method for the further implementation. The key expected outcomes are optimum cutting speed, feed rate, and depth-of-cut for the lowest surface roughness parameters and cutting temperature.

## 2. Materials and Method

The hard-turning operation was conducted by machining of hardened high-carbon steel i.e., AISI 1060 in a center lathe (Origin: SJR Machinery Co., Ltd., Nantong, China, Max. W/P length: 1 m). The material stock had dimensions of length 300 mm and diameter 100 mm. The cutting tool used was coated tungsten carbide (WC) insert with Chemical Vapor Deposition (CVD) coating with TiCN/Al_2_O_3_/TiN. The insert’s ISO designation was SNMM 120408. When the insert was used with the tool holder PSBNR 2525M12 (Sandvik Coromant, Sandviken, Sweden), the tool cutting edge was 75°, the clearance angle was 6°, and orthogonal rake angle was −6°. The hardening of work material was performed by austenizing followed by oil quenching and, lastly, tempering. The temperatures for the respective stages were 900 °C, 30 °C, and 370 °C. After the heat treatment, the hardness of the workpiece was 40 ± 2 Rockwell C.

The machining runs were varied according to the variations made in machining parameters. Investigated machining parameters were cutting speed, feed rate, and depth-of-cut. The Taguchi L8 orthogonal array was used for the design of experiment (DOE) to reveal 8 experiments (Table 1) based on 4 levels of cutting speed, and 2 levels of feed and cutting depth. This DOE reduced the number of experiments by 50% compared to full factorial DOE—thus, a step towards conservation of resources. The selection of machining parameters values are based on the knowledge of literature and current industrial practice.

The representative indices of surface quality, two surface roughness parameters, were recorded after each machining run. Those parameters were (i) arithmetic mean deviation of surface roughness, *R_a_*, and (ii) maximum height of profile of surface roughness, *R_z_*. Their measurement was conducted by using SRG 4500 roughness tester (cut-off length 0.8 mm, Thread Check Inc., New York, NY, USA). The cutting temperature was measured by using a tool–work thermocouple. Initially, the mili-volt reading of the thermocouple was recorded, and after that the mili-volt value was converted into temperature in Celsius scale. Proper calibration was done before using the thermocouple. For details on temperature measurement by tool–work thermocouple, refer to authors’ other work [16]. The measured responses are listed in Table 1.

## 3. Intelligent Optimization Algorithms

### 3.1. Teaching–Learning-Based Optimization (TLBO)

The TLBO, reported by Rao et al. [7], is a population-based optimization method that mimics the behavior of teachers and learners by which process the teachers teach and the learners learn. This algorithm considers the teachers and learners as two fundamental components. Herein, the learning is accomplished in two forms: (i) learning from teachers and (ii) learning from other learners. The first one is called the teacher phase while the second one is named the learner phase. Evidently, the performance of the algorithm was evaluated by the grades of the learners which in turn is dependent on the quality of the teachers. Note that the learners are regarded as population and the subjects offered by the teachers are design parameters and the achieved grades are the ‘fitness’ value. Both phases are discussed below.

Teacher phase: In this section, the learners learn from the teachers only. Here, the quality of teachers influences the learning outcomes. As to be noted, the best quality learners are assigned as the teachers. The objective of teachers is to increase the mean results of the class. For instance, consider that in iteration “*i*” the numbers of subjects taught are “*m*”, size of learners is “*n*” numbers, for specific subject “*j*” the mean of the result is *M_j,i_*. Now the result of best learner *k_best_* can be considered as the overall best result *X_total-kbest-i_*. In deciding this best result, the whole subject spectrum is accounted. The learner with this best result is usually considered as teacher. The difference of the mean of the learners of a subject and the best learner (i.e., teacher) can be presented by Equation (1) [7].

(1) $$Diff\_Mean_{j,k,i}=r_{i}\left(X_{j,kbest,i}-T_{F}M_{j,i}\right)$$

where 0 ≤ *r_i_* ≤ 1 is random number and the *T_F_* indicates the teaching factor that to be determined by Equation (2) [7].

(2) $$T_{F}=round\left[1+rand\left(0,1\right)2-1\right]$$

where the distribution *T_F_* follows equal probability distribution and it is not considered as parameter of TLBO.

The current solution is updated by the Equation (3) [7].

(3) $$X_{j,k,i}^{\prime }=X_{j,k,i}+Diff\_Mean_{j,k,i}$$

In this manner, the updated result is accepted if it is better. At the last stage of teacher phase, the updated results are saved and used as the input to the learner phase.

**Figure 1:**
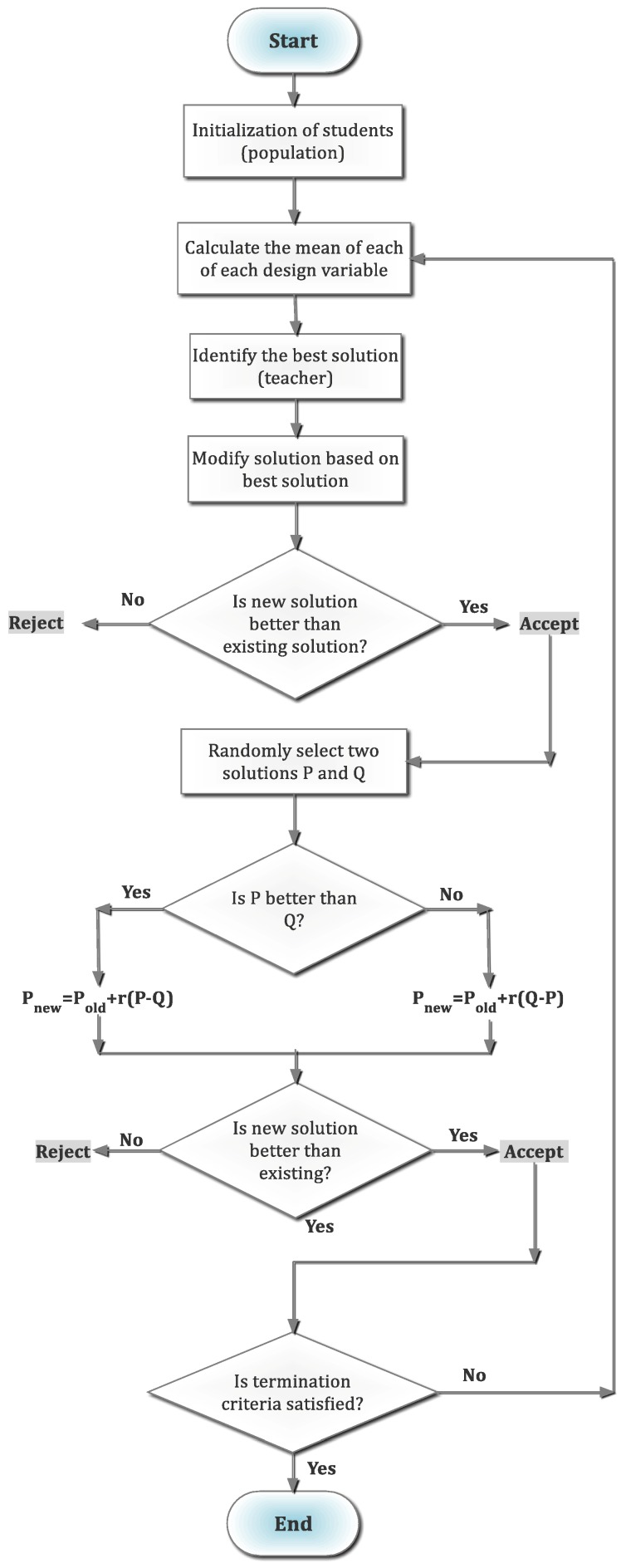
Flowchart for teaching—learning-based optimization (TLBO).

Learner phase: In this stage, the learners enhance the learning by the effective interaction among the other learners. In a random manner, one learner learns from another learner only if that learner has better knowledge than him/her as defined in Equations (4) and (5) [7]. As mentioned, the two random learners are *P* and *Q*, who had results $X_{total-P,i}$ and $X_{total-Q,i}$, have updated results $X_{total-P,i}^{\prime }$ and $X_{total-Q,i}^{\prime }$ at the end of the teacher phase. However, the $X_{total-P,i}^{\prime }$ ≠ $X_{total-Q,i}^{\prime }$.

(4) $$X_{j,P,i}^{\prime \prime }=X_{j,P,i}^{\prime }+r_{i}\left(X_{j,P,i}^{\prime }-X_{j,Q,i}^{\prime }\right)\;\mathrm{If}\;X_{total-P,i}^{\prime }<X_{total-Q,i}^{\prime }$$

(5) $$X_{j,P,i}^{\prime \prime }=X_{j,P,i}^{\prime }+r_{i}\left(X_{j,Q,i}^{\prime }-X_{j,P,i}^{\prime }\right)\;\mathrm{If}\;X_{total-Q,i}^{\prime }<X_{total-P,i}^{\prime }$$

where the $X_{total-P,i}^{\prime \prime }$ is accepted when it has better result. After the learner stage, all the better results are saved and afterward used as inputs to the next iteration of teacher phase. The flow chart of TLBO is demonstrated in Figure 1.

### 3.2. Bacteria Foraging Optimization (BFO)

In recent times, the adaptation of the bacteria foraging optimization method [17], primarily inspired from the attitudes of E. Coli’s, is notably reported with significant success. This method is considered as an intelligent method that is meta-heuristic in nature. The nature of response of the bacteria with respect to changes in the surrounding environment, especially in the chemical gradient, stands out as the backbone of this method. In concept, the movement of the bacteria cells i.e., agents is driven by the awareness for food in the environment. In this manner, the cells are moved towards the optimal condition. For ease of understanding, the procedure of BFO is listed here.

Chemotaxis: The representative step for the behaviors like swimming as well as tumbling. In this step, the basic mechanism is to search the nutrients in any random directions. At any point, when the nutrient gradient is run into, the behavior of bacteria is dominated as the swim rather than tumble. Henceforth, the chemotaxis can be expressed as:

Function $\theta^{i}\left(j,k,l\right)$ represents the position of $i^{th}$ bacterium that possesses the $j^{th}$ chemotaxis having $k^{th}$ reproduction and $l^{th}$ elimination and dispersal. Also, mathematically the directional adjustment is modified according to Equation (6) [17].

(6) $$\theta \left(j+1,k,l\right)=\theta \left(j,k,l\right)+C\left(i\right)\cdot \frac{\phi \left(i\right)}{\sqrt{\phi^{T}\left(i\right)\cdot \phi \left(i\right)}}$$

Here, the success of the optimization is largely dependent on the traits of foraging process of bacteria.

Swarming: In this stage, the randomly moved bacteria are organized in sophisticated uniformity as colonies. This altogether swarming is caused by the signals given by the cell, and it is mathematically presented by Equation (7) [17].

(7) $$J_{CC}\left(\theta ,P\left(j,k,l\right)\right)=\overset{S}{\underset{i=1}{\sum }}J_{CC}{}^{i}\left(\theta ,\theta \left(j,k,l\right)\right)\;=\overset{s}{\underset{i=1}{\sum }}\left[-d_{attract}exp\left(-\omega_{attract}\overset{P}{\underset{n=1}{\sum }}{\left(\theta_{n}-\theta_{n}^{i}\right)}^{2}\right)\right]\;+\overset{s}{\underset{i=1}{\sum }}\left[-d_{repellent}exp\left(-\omega_{repellent}\overset{p}{\underset{n=1}{\sum }}{\left(\theta_{m}-\theta_{n}^{i}\right)}^{2}\right)\right]$$

In Equation (2), the value of cost function is denoted by $J_{cc}\left(\theta ,P\left(j,k,l\right)\right)$. Note that the addition of varying cost function to the value of cost function results in actual cost function—that needs to be minimized. Furthermore, the *S* and *P* represent the numbers of total bacteria and parameters for optimization respectively. The parameters such as *d_attract_*, *w_attract_*, *d_repellent_*, *w_repellent_* need to be selected correctly for each bacterium.

Reproduction: The health condition of the bacteria is defined by Equation (8) [17]. This phase is characterized by the reproduction of comparatively better-fitted bacteria into two bacteria. Note that the least-fitted bacteria die; as such, the overall population remains constant.

(8) $$J_{health}^{i}=\overset{N_{c}+1}{\underset{j=1}{\sum }}J\left(j,k,l\right)$$

Elimination and dispersion: In case of scarcity of bacteria in any place, the bacteria of other places may face dispersal. This is due to the changing nature of habitable environment of the bacteria. In fact, such dispersal may cause destruction of chemotactic process. It is also possible that the dispersal causes the chemotaxis process to be assisted by adequate nutrient sources in proximity.

**Figure 2:**
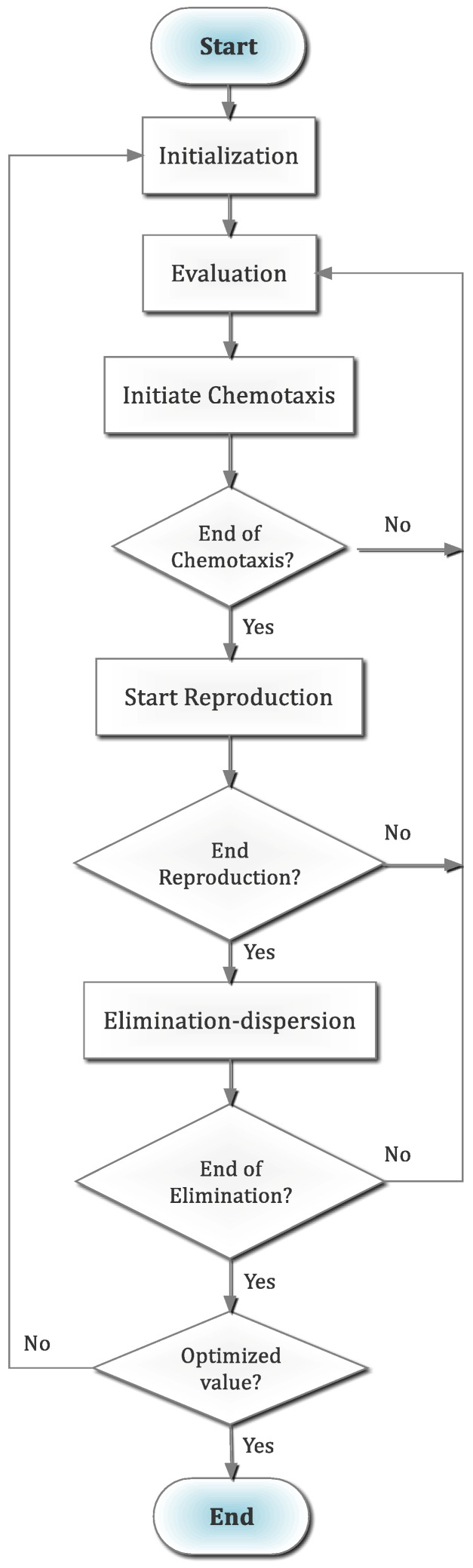
Flowchart for bacteria foraging optimization.

In the above fashion, the bacteria is never satisfied with the nutrients they get, and thereby they keep searching—it indicates the continuous nature of the chemotaxis, swarming, reproduction, and elimination and dispersal steps. The flow chart of BFO is shown in Figure 2.

## 4. Results and Discussion

Initially, the effects of the control factors i.e., cutting speed, feed rate, and depth-of-cut on the responses are portrayed graphically and discussed to understand the role of factors on the responses. Then, the surface-roughness parameters and cutting temperature found in hard turning were optimized by using the teaching–learning-based optimization and bacteria foraging optimization separately. Later, the optimum results are compared with respect to common parameters of interest. Eventually, the best optimization method is selected.

### 4.1. Results

**Figure 3:**
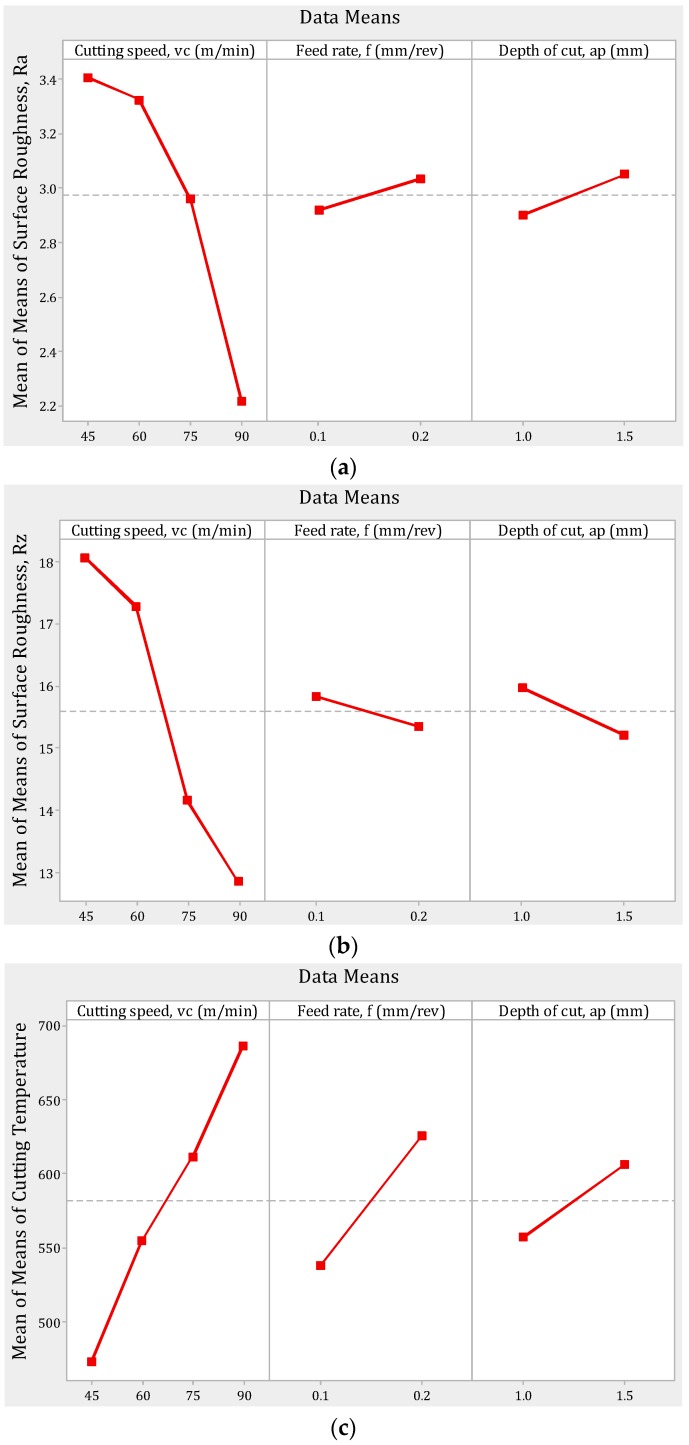
Effects of control factors on (**a**) mean of surface roughness, *R_a_*, (**b**) mean of surface roughness, *R_z_*, and (**c**) mean of cutting temperature.

The hard-turning operation is controlled by three factors—namely the cutting speed, feed rate, and depth-of-cut. Hence, these parameters mostly influence the eventual outcomes of machining. For that reason, the mean behaviors of the surface-roughness parameters as well as the cutting temperature are plotted in Figure 3. It is evident from Figure 3a that the increase in cutting speed is reflected by a decrease in the surface roughness, *R_a_*. This is due to the fact the lower cutting speed is associated with increased chatter of machine tool. Moreover, the higher cutting speed is associated with lower coefficient of friction. In addition, the increased cutting speed causes the temperature to rise (Figure 3c), which, in turn, softens the material. As such, an ease of cutting is experienced [18]. A similar movement of surface roughness *R_z_* with respect to cutting speed is also noticeable in Figure 3b. Note that the increase in cutting temperature with the increase in cutting speed is due to the conversion of mechanical energy (rotation of job in spindle) into heat energy.

The effects of feed rate on the surface-roughness parameters were opposing in nature (Figure 3a,b). For instance, an increase in feed rate caused a slight increase in *R_a_* (this is according to the theoretical relation of *f* and *R_a_*) while causing a slight decrease in *R_z_*. Similarly, when the depth of cut was increased, the *R_a_* increased by a slight amount but the *R_z_* deceased by a small amount. This opposing nature can be interpreted by the fact that *R_a_* is the average parameter of roughness while the *R_z_* is the maximum height of roughness. As such, it is possible to get lower *R_z_* when the *R_a_* is increasing. Lastly, the increase in all three factors caused an increase in the cutting temperature. This is expected by the fact that the material removal rate (MRR) = *v_c_*·*f*·*a_p_*. This means an increase in speed, feed, and depth causes an increase in the amount of materials removed per unit of time. When the MRR increases, more energy is required to deform such increased material. Consequently, the thermal state of cutting zone experiences higher temperature.

It is also appreciable that the cutting speed has the highest contribution on both the surface-roughness parameters and cutting temperature. Compared to cutting speed, the feed rate and depth-of-cut have minor roles in defining the value of roughness and temperature. Hence, during optimization, the change in cutting speed is most significant to favorably align the value of the responses.

### 4.2. Optimization by TLBO and BFO

Before optimization, the two roughness parameters and the cutting temperature were converted into a single function (normalized) as shown in Equation (9).

(9) Min Z=W1(RaRamin)+W2(RzRzmin)+W3(θθmin)

where the *W*_1_, *W*_2_, and *W*_3_ are weight factors for average surface roughness parameter, maximum height surface-roughness parameter, and cutting temperature, respectively. However, their summation should be 1.0. Also, the ${R_{a}}_{min}$ and ${R_{z}}_{min}$ are the minimum values of the average surface roughness parameter and maximum height surface roughness parameter respectively found in hard turning. For this current study, all three responses were considered to possess equal weight (*W*_1_ = *W*_2_ = *W*_3_ = 1/3) as all of them are valuable to the manufacturers as they largely influence the machining outcomes.

The software used for performing the optimization was Matlab 2018b (The MathWorks, Natick, MA, USA) with intel i5 Processesor and 4 GB RAM. By nature, TLBO depends on the population size and the generation numbers which makes this algorithm parameter-less. To make the solution, the trials runs are conducted that eventually caused the population size to be 50 and generation to be 100. On the other hand, in the BFO method, the optimization was started with the initial parameters listed in Table 2. Initially, the 50 bacterial elements were taken into account to run the algorithm.

The optimum results by the TLBO and BFO methods are listed in Table 3. It is visible that the cutting speed of 80 m/min was fixed as the optimum cutting speed. Interestingly, from Figure 3, it is observable that the highest cutting speed (90 m/min) was responsible for the lowest surface roughness; however, at the same time, it caused the temperature to be highest. At this point, a trade-off is required to make the system congenial for both the surface roughness and the cutting temperature. And, because of this reason, the TLBO reported 80 m/min as the optimum cutting speed. For the same opposing nature (discussed earlier in Figure 3), the feed rate of 0.13 mm/rev was found as the optimum feed rate. However, the highest value of depth of cut (1.5 mm) was the optimum depth-of-cut. On other side, the BFO approach, within 16 s, revealed the optimum run. The optimum parameters by BFO are a cutting speed of 75 m/min, a feed rate of 0.10 mm/rev, and a depth-of-cut of 1.3 mm.

**Figure 4:**
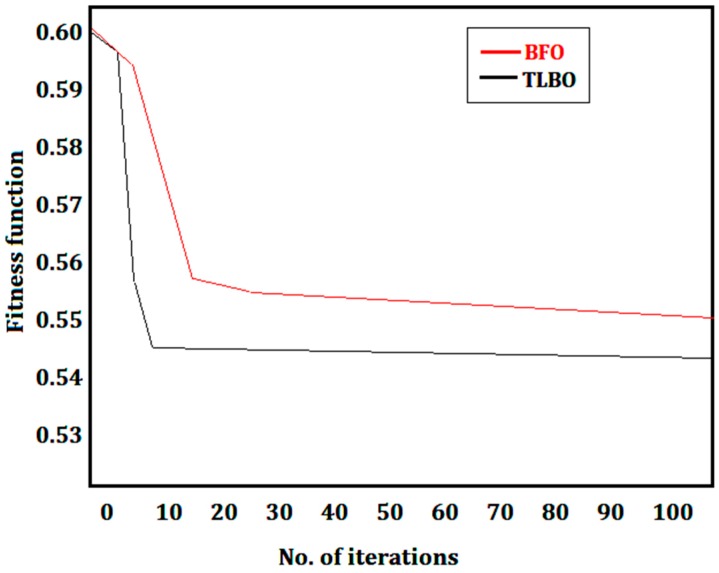
Convergence of bacteria foraging optimization (BFO) and teaching–learning-based optimization (TLBO).

A comparison of TLBO and BFO revealed that the best solution was given by the TLBO method (*Z* is minimum = 0.54326). Further, this solution was found within the lowest time—for TLBO the time was 4 s while that for BFO was 16 s—and four times faster than the BFO. The convergence of the TLBO and BFO is illustrated in Figure 4. With the number of iterations, the overall fitness function of the TLBO aligns better to the optimum fitness function value. Hence, between the TLBO and BFO, the TLBO is recommended.

## 5. Conclusions

In this study, two intelligent optimization algorithms were employed for the optimization of hard-turning parameters. Adoption of evolutionary optimization methods, with the assistance of high-level computing, can convert the conventional machining processes to be more effective, efficient, and cost-economic. From this study, the following conclusions can be drawn:

- Intelligent optimization is an important ingredient of smart manufacturing in which the learning capability of the method is required—which is present in both teaching–learning-based optimization and bacteria foraging optimization. Lack of implementation of these methods in hard turning motivated the current study, and eventually their successful implementation is shown here.
- The influences of cutting speed, feed rate, and cutting depth on the arithmetic mean deviation of surface roughness *R_a_*, the maximum height of the profile of surface roughness *R_z_,* and cutting temperature are investigated by portraying the main effects plot. It was found that the cutting speed played the most dominant role in defining the roughness parameter as well as the temperature. Moreover, an increase in cutting speed resulted in a decrease in the roughness values but an increase in the cutting temperature. This outcome necessitated a trade-off of factor values.
- Trade-off of the responses/factors was accomplished by employing the intelligent optimization method i.e., TLBO and BFO. Optimum results by the TLBO approach were a cutting speed of 80 m/min, feed rate of 0.13 mm/rev, and depth-of-cut of 1.5 mm; optimum parameter settings by BFO were a cutting speed of 70 m/min, feed rate of 0.10 mm/rev and depth-of-cut of 1.3 mm.
- The TLBO was found to be superior to the BFO in terms of better convergence and shorter time of computation—hence, the TLBO is recommended.
- Future research direction can be the adoption of evolutionary methods in the parametric optimization of additive manufacturing processes. Also, further research attention can be given to the integration of optimization methods with the real-time parameter optimization.

## Figures and Tables

**Table 1:** Taguchi L8 orthogonal array and values of responses found from machining runs.

Experiment Number	Cutting Speed, *v_c_* (m/min)	Feed Rate, *f* (mm/rev)	Depth of Cut, *a_p_* (mm)	Surface Roughness, *R_a_* (µm)	Surface Roughness, *R_z_*(µm)	Cutting Temperature, *θ* (°C)
1	45	0.1	1.0	2.60	14.36	404
2	45	0.2	1.5	4.21	21.75	543
3	60	0.1	1.0	3.87	22.20	488
4	60	0.2	1.5	2.78	12.35	622
5	75	0.1	1.5	3.51	16.48	585
6	75	0.2	1.0	2.41	11.85	638
7	90	0.1	1.5	1.70	10.26	674
8	90	0.2	1.0	2.73	15.45	699

**Table 2:** Input parameters in the bacteria foraging optimization (BFO) algorithm.

Parameters	Values
Number of bacterial elements considered, *S*	50
Max defined chemotactic steps, *N_c_*	50
Max defined reproduction steps, *N_re_*	4
Total elimination–dispersal event, *N_ed_*	2
Max allowed swim steps, *N_s_*	4
Elimination–dispersal probability, *P_ed_*	0.1

**Table 3:** Comparison of TLBO and BFO.

Parameters	TLBO	BFO
Cutting speed (m/min)	80	75
Feed rate (mm/rev)	0.13	0.10
Depth of cut (mm)	1.5	1.3
Best solution (minimum of *Z*)	0.54326	0.55262
Worst solution	0.56592	0.57854
Average time (s)	4 s	16 s
